# A Pilot Study on Donor Human Milk Microbiota: A Comparison with Preterm Human Milk Microbiota and the Effect of Pasteurization

**DOI:** 10.3390/nu14122483

**Published:** 2022-06-15

**Authors:** Isadora Beghetti, Monica Barone, Luigia De Fazio, Eleonora Laderchi, Elena Biagi, Silvia Turroni, Patrizia Brigidi, Andrea Pession, Luigi Corvaglia, Arianna Aceti

**Affiliations:** 1 Department of Medical and Surgical Sciences, University of Bologna, 40138 Bologna, Italy; isadora.beghetti@studio.unibo.it (I.B.); monica.barone@unibo.it (M.B.); luigia.defazio@gmail.com (L.D.F.); eleonora.laderchi@studio.unibo.it (E.L.); patrizia.brigidi@unibo.it (P.B.); andrea.pession@unibo.it (A.P.); luigi.corvaglia@unibo.it (L.C.); 2 Neonatal Intensive Care Unit, IRCCS Azienda Ospedaliero Universitaria di Bologna, 40138 Bologna, Italy; 3 Unit of Microbiome Science and Biotechnology, Department of Pharmacy and Biotechnology, University of Bologna, 40126 Bologna, Italy; elena.biagi@unibo.it; 4 Paediatric Unit, IRCCS Azienda Ospedaliero Universitaria di Bologna, Via Massarenti 11, 40138 Bologna, Italy

**Keywords:** very low birthweight preterm infants, human milk microbiota, donor human milk, pasteurization

## Abstract

Human milk (HM) is the best feeding option for preterm infants; however, when mother’s own milk (MOM) is not available, pasteurized donor human milk (DHM) is the best alternative. In this study, we profiled DHM microbiota (19 samples) using 16S rRNA amplicon sequencing and compared its compositional features with the MOM microbiota (14 samples) from mothers who delivered prematurely (PT-MOM). As a secondary study aim, we assessed the specific effect of pasteurization on the characteristics of the DHM microbiota. DHM showed significantly higher alpha diversity and significant segregation from PT-MOM. Compositionally, the PT-MOM microbiota had a significantly higher proportion of Staphylococcus than DHM, with Streptococcus tending to be and Pseudomonas being significantly overrepresented in DHM compared with the PT-MOM samples. Furthermore, pasteurization affected the HM microbiota structure, with a trend towards greater biodiversity and some compositional differences following pasteurization. This pilot study provided further evidence on the HM microbial ecosystem, demonstrating that the DHM microbiota differs from the PT-MOM microbiota, possibly due to inherent differences between HM donors and mothers delivering prematurely, and that pasteurization per se impacts the HM microbiota. Knowledge about HM microbiota needs to be acquired by investigating the effect of DHM processing to develop strategies aimed at improving DHM quality while guaranteeing its microbiological safety.

## 1. Introduction

Infants born prematurely possess a fragile gut microbiota that is particularly vulnerable to perturbations [1]. Several prenatal, neonatal, and postnatal factors negatively impact the developmental trajectory of the gut microbiota of the preterm infant [2]. The resulting alterations have been linked to life-threatening diseases such as necrotizing enterocolitis (NEC) and late-onset sepsis (LOS) and may impact future risk of asthma, atopy, obesity, and psychosocial diseases [3,4,5].

Breastfeeding is considered the gold standard for infants’ nutrition, as mother’s own milk (MOM) provides all the nutritional factors required for optimal infant development and contains a variety of bioactive factors that promote a healthy assembly of gut microbiota [6]. Encouragingly, human milk (HM) also contains a specific microbiota that serves as a source of potentially probiotic bacteria [7,8,9,10]. Once in the infant gut, these bacteria participate in the physiological development of the gut microbiota and may contribute to infant metabolism, protection against infection, and neuro- and immune modulation by means of direct and indirect host–microbiota cross talk [11,12].

HM feeding is of utmost importance for preterm infants. The provision of exclusive MOM is linked to a reduction in the incidence of NEC and LOS, as well as to improved neurodevelopment [13]. However, mothers of preterm infants are frequently unable to produce enough milk to meet their infants’ needs. Therefore, donor human milk (DHM) is widely recommended over formula whenever MOM is insufficient or not available [14]. To ensure its quality and safety, DHM must be provided by a qualified HM bank (HMB) [15]. DHM must be pasteurized to inactivate potentially harmful viral and bacterial agents. However, pasteurization impairs several nutritional and biological HM properties [16,17] and is very likely to interfere with the resident HM microbiota, thus compromising the probiotic effect of MOM. On the other hand, it must be said that according to recent evidence, the health benefits of some microbes may not necessarily rely on their viability [18]. In analogy with “paraprobiotics”, “ghost probiotics”, or, as recently defined, “postbiotics” (i.e., a preparation of inanimate microorganisms and/or their components that confers a health benefit on the host) [19], it could be argued that DHM processed through pasteurization could still harbor a ghost microbiota capable of positively influencing host health. However, to date, little has been reported about DHM microbiota composition, and limited information is available about variations in the diversity of HM microbiota following pasteurization and its potential biological significance [20].

In an attempt to bridge this gap, here we profiled the DHM microbiota with 16S rRNA amplicon sequencing and compared its compositional features with the MOM microbiota from mothers who delivered prematurely. As a secondary study aim, we assessed the specific effect of pasteurization on the characteristics of the DHM microbiota.

## 2. Materials and Methods

### 2.1. Experimental Design

A pilot observational study was conducted between May 2020 and January 2021 at the Neonatal Intensive Care Unit (NICU) of IRCCS AOU Bologna, Italy. For the primary study aim, HM samples were collected from mothers who delivered prematurely (PT-MOM samples) and whose infants were admitted to the NICU between the second/third day and the third week after delivery. During the same period, DHM samples were collected from the HMB of Bologna. The microbiota features of the DHM samples were compared with those of the PT-MOM samples. In addition, further HM samples from HM donors were collected and analyzed for microbiota profiles before (raw HM [RHM]) and after pasteurization (pasteurized HM [PHM]).

### 2.2. Subject Recruitment

Mothers who delivered prematurely between May 2020 and January 2021 at the NICU of IRCCS AOU Bologna (Italy) were included in the study. Eligibility criteria were: (i) delivery at gestational age <32 weeks and/or newborn’s birth weight ≤1500 g; (ii) maternal age ≥18 years; and (iii) preterm infant receiving an exclusive HM diet (PT-MOM and/or DHM). The exclusion criteria included ongoing maternal infection or severe clinical conditions. Women recruited as HM donors at the Bologna HMB in the same period were also enrolled. In order to participate in the study, donors had to fulfill the criteria set by the Italian Donor Human Milk Bank Association for milk donation [21]. Descriptive data related to the clinical demographic characteristics of the mothers who delivered prematurely, mothers recruited as HM donors and their respective newborns were collected.

The study was conducted in conformity with the principles and regulations of the Helsinki Declaration. The study protocol was approved by the Institutional Ethical Committee (CE-AVEC)—study ID 474/2019/Sper/AOUBo. Preterm infants’ mothers and HMB donors were required to provide written informed consent to participate in the study. Demographic and clinical data were recorded in a specific case report form.

### 2.3. Milk Samples Collection and Processing

#### 2.3.1. Donor HM Collection

Donors of the HMB of Bologna who took part in the present study were recruited within 3 months of delivery, following usual HMB procedure. DHM was collected at home by mothers after accurate hand washing using hand sanitizer and breast washing. The collection of all DHM samples was performed with the aid of an electric breast pump connected to sterile disposable collection kits into sterile ad hoc bottles, in accordance with the guidelines for HM collection used in HMB. Approximately 100 mL of HM was collected from each donor. Donor HM was stored at donors’ home in a refrigerator until delivery to the HMB, maintaining the cold chain. DHM samples were stored at −20 °C at the HMB until further processing and then handled following the routine protocol for DHM. In particular, DHMs were thawed, pooled and then pasteurized through Holder pasteurization (HoP). For the secondary aim of the study (i.e., the effect of pasteurization on the DHM microbiota), additional 100-mL MOM samples were collected from donors in accordance with the guidelines for HM collection used in HMB and subdivided into two aliquots in sterile tubes. The first aliquot was not heat treated (RHM) and was stored at −20 °C, while the second aliquot was processed by HoP (PHM). HoP, currently recommended for use in HMB, was performed at the Bologna HMB, following the standard pasteurization procedure for DHM used in our NICU [21,22]. Specifically, a standard HM Holder pasteurizer (S90 TES, Medicare Colgate Ltd., Cullompton, UK) was used, and the bottled DHM was pasteurized using a temperature of 62.5 °C for 30 min (tolerance ± 0.5 °C); then it was cooled to 4 °C in 60 min (tolerance ± 0.5 °C) [23].

#### 2.3.2. Preterm–Delivering Mother’s Own Milk Collection

Preterm–delivering mothers (PTMs) followed the same personal hygiene instructions given to HMB donors. Each HM sample was collected into a sterile bottle using an electric breast pump of the hospital breastfeeding room and a personal breast pump kit. The breast pump kit had to be cleaned and sterilized before every collection. Between the second/third day and the third week post-delivery, enrolled PTMs collected a sample of 50 mL of their own fresh milk. After collection, all PT-MOM samples were immediately frozen at −20 °C and delivered to the laboratories for further analyses.

### 2.4. Microbiota Profiling

#### 2.4.1. Microbial DNA Extraction

Microbial DNA was extracted from the milk samples using the DNeasy PowerSoil kit (QIAGEN, Hilden, Germany), following the manufacturer’s instructions along with the modifications previously described by Douglas et al. [24]. Briefly, 1.5 mL of HM was pelleted by centrifugation at 13,000 rpm at 4 °C for 20 min. The fat layer was removed along with the liquid supernatant. The cell pellet was directly resuspended in bead solution and added to a PowerLyzer Glass Bead Tube with C1 solution before incubation at 65 °C for 10 min. Samples were homogenised using FastPrep-24 (MP Biomedicals, Irvine, CA, USA) for 3 cycles of 1 min at 6.5 movements/s. DNA yield was assessed using Qubit 3.0 fluorometer (Life Technologies, Waltham, MA, USA).

#### 2.4.2. 16S rRNA Amplicon Sequencing

For each sample, the V3-V4 region of the 16S rRNA gene was amplified using S-D-Bact-0341-b-S-17/S-D-Bact-0785-a-A-21 primers with Illumina overhang adapter sequences. PCR reactions were performed in a final volume of 25 μL, containing 12.5 ng of genomic DNA, 200 nM of each primer, and 2X KAPA HiFi HotStart ReadyMix (Kapa Biosystems, Roche, Basel, Switzerland) in a Thermal Cycler T (Biometra GmbH, Göttingen, Germany) with the following gradient: 3 min at 95 °C for the initial denaturation, 25 cycles of denaturation at 95 °C for 30 s, annealing at 55 °C for 30 s and extension at 72 °C for 30 s, and a final extension step at 72 °C for 5 min. PCR products were purified using a magnetic bead system (Agencourt AMPure XP, Beckman Coulter, Brea, CA, USA), indexed by limited-cycle PCR using Nextera technology, and further purified. Final libraries were pooled at equimolar concentrations, denatured with 0.2 N NaOH, and diluted to 6 pM prior to loading onto the MiSeq flow cell for sequencing with the 2 × 250 bp paired-end protocol, per manufacturer’s instructions (Illumina, San Diego, CA, USA). Sequencing data are available at NCBI SRA under the BioProject ID PRJNA849176.

### 2.5. Bioinformatics and Statistics

Raw sequences were processed using a pipeline combining PANDAseq [25] and QIIME 2 [26]. DADA2 [27] was used to bin high-quality reads into amplicon sequence variants (ASVs). Taxonomy was assigned using VSEARCH [28] and the Greengenes database as a reference. Alpha diversity was assessed using the Shannon and inverse Simpson indices. Beta diversity was evaluated using the Bray-Curtis dissimilarity measure.

All statistical analysis was performed in R 3.3.2, using RStudio 1.0.44 and the libraries vegan (http://www.cran.r-project.org/package=vegan/, accessed on 14 February 2021) and made4 [29]. The significance of data separation in the principal coordinates analysis (PCoA) of Bray-Curtis distances was tested using a permutation test with pseudo-*F* ratio (function adonis of vegan). The nonparametric Wilcoxon test was used to assess significant differences between groups for intra-sample diversity and relative taxon abundances. All *p*-values were corrected for false discovery rate (FDR, Benjamini-Hochberg) [30], and *p*-values ≤ 0.05 were considered statistically significant, while *p*-values ≤ 0.1 were considered a trend.

Clinical data distribution was verified using the Shapiro-Wilk test. Continuous variables were expressed as median (interquartile range [IQR]). Clinical characteristics were compared between PTMs and HMB donors using Fisher’s exact test for categorical variables and the Mann-Whitney U test for continuous variables. Statistical Package of Social Science (SPSS, IBM, Chicago, IL, USA) software, version 26, was used for clinical statistical analysis, and *p*-values ≤ 0.05 were considered statistically significant.

## 3. Results

### 3.1. Clinical Characteristics of the Study Population

A total of 7 PTMs were enrolled in the study and 14 PT-MOM samples were collected, 2 from each mother. The median age was 32 (IQR, 28–34) years. Two PTMs were primiparae; five PTMs were Caucasian, the other two were Asian. The neonates had a median gestational age of 31.6 (IQR, 27.9–32.3) weeks and a median weight of 1245 (IQR, 1022–1633) g. Most were delivered by caesarean section (85.7%, *n* = 6); two were planned caesarean sections, the other four were emergency caesareans. The only vaginal delivery followed a preterm prolonged rupture of membranes. No infant was directly breastfed during the study period. All infants were tube and/or bottle-fed.

Fourteen donors from the Bologna HMB were included in this study. One DHM sample was available for each donor, while two samples were available for five donors. The median gestational age was 40 (IQR, 39.4–40.4) weeks. The median age was 34 (IQR, 30.7–36.2) years, and two donors were primiparae. Thirteen donors were Caucasian, while one was Asian. No differences in age and parity were observed between PTMs and HMB donors, but PTMs showed a higher rate of caesarean section and lower gestational age at delivery (Table 1).

### 3.2. Human Milk Microbiota Profiling

Microbial DNA extracted from a total of 14 PT-MOM and 19 DHM samples underwent 16S rRNA gene Illumina sequencing. A total of 1,103,438 sequencing reads (range, 12,689–181,071), clustered in 2846 ASVs, were obtained and analysed.

#### 3.2.1. PT-MOM and DHM Microbiota

**Figure 1:**
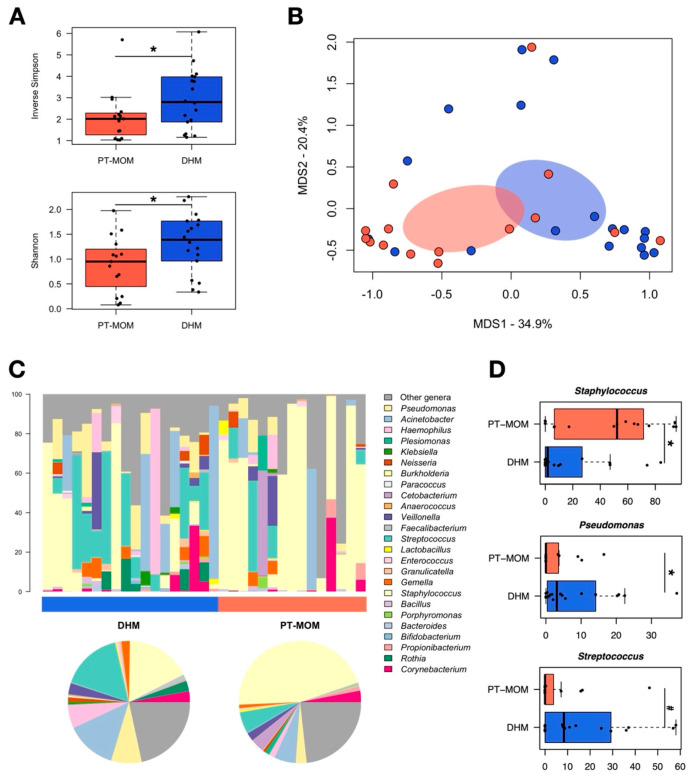
Milk microbiota from preterm-delivering mothers compared with donor human milk microbiota. (**A**) Boxplots showing the distribution of alpha diversity, assessed by the inverse Simpson (**top**) and Shannon (**bottom**) indices, in the milk microbiota from preterm-delivering mothers (PT-MOM) and donor human milk microbiota (DHM). *, *p* value ≤ 0.05, Wilcoxon test. (**B**) Principal coordinate analysis (PCoA) of the Bray-Curtis distances between the genus-level microbial profiles of the two study groups, PT-MOM (salmon) and DHM (blue). A significant separation was found (*p* = 0.02, permutation test with pseudo-*F* ratio). (**C**) Genus-level relative abundance profiles of PT-MOM and DHM samples. Data are shown in the bar charts for each sample and in the pie charts as mean values in the study groups. (**D**) Boxplots showing the relative abundance distribution of bacterial genera significantly differentially represented between groups. *, *p* ≤ 0.05, Wilcoxon test. For Streptococcus, only a trend was found (#, *p* = 0.08).

First, the PT-MOM microbiota was compared with that of DHM. According to the Shannon and inverse Simpson indices, alpha diversity was significantly higher in DHM than in PT-MOM (*p* ≤ 0.05) (Figure 1A). As for inter-sample variability, the PCoA analysis based on the Bray-Curtis dissimilarity showed a significant segregation between the two groups (*p* = 0.02) (Figure 1B). Compositionally (Figure 1C,D), the PT-MOM microbiota was largely dominated by *Staphylococcus* (mean relative abundance ± SEM, 45.4 ± 9.5%), whose proportions were significantly higher than in the DHM microbiota (*p* = 0.02). On the other hand, the latter was jointly dominated by *Streptococcus*, *Staphylococcus*, and *Acinetobacter* (17.4 ± 4.5%, 16.6 ± 6.1%, and 12.6 ± 5.7%, respectively), with *Streptococcus* tending to be overrepresented compared with PT-MOM samples (*p* = 0.08). Furthermore, the PT-MOM microbiota was comparatively depleted in *Pseudomonas* (*p* = 0.03).

#### 3.2.2. Impact of Pasteurization on the HM Microbiota

**Figure 2:**
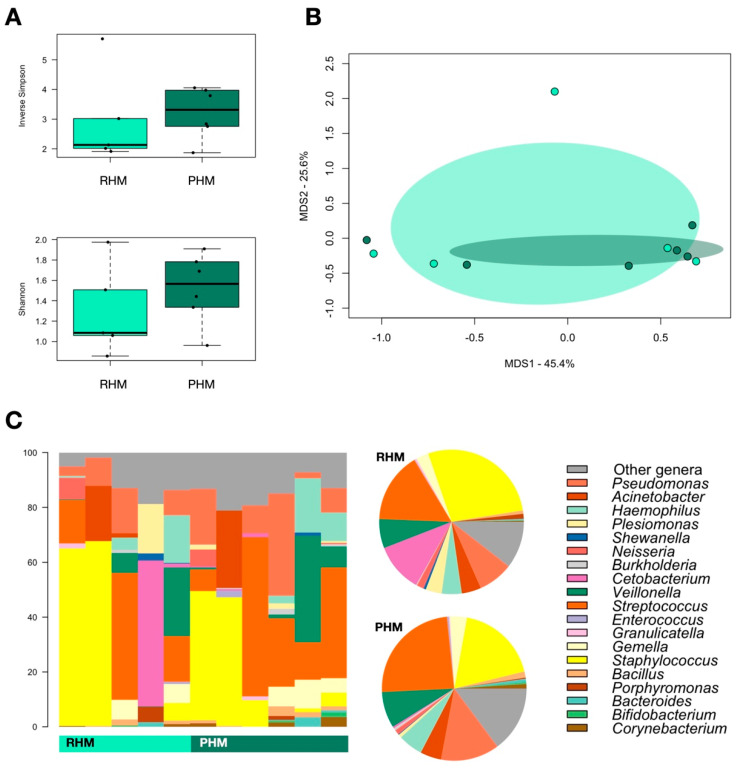
The impacts of pasteurization on donor human milk microbiota. (**A**) Boxplots showing the distribution of alpha diversity, assessed by the inverse Simpson (**top**) and Shannon (**bottom**) indices, in donor human milk microbiota before (RHM) and after pasteurization (PHM). (**B**) Principal coordinate analysis (PCoA) of the Bray-Curtis distances between the genus-level microbial profiles of the two study groups, RHM (light green) and PHM (green). (**C**) Genus-level relative abundance profiles of RHM and PHM samples. Data are shown in the bar charts for each sample and in pie charts as mean values in the study groups.

Next, we explored HoP-induced changes in the HM microbiota by comparing the profiles of six additional DHM samples collected from 3 HMB donors before (RHM) and after pasteurization (PHM). Albeit in the absence of statistical significance, HoP resulted in an increase in alpha diversity (Figure 2A) and tended to reduce beta diversity (Figure 2B). At the taxonomic level (Figure 2C), there were no significant differences between the RHM and PHM samples. However, it is worth noting that in PHM, the relative abundance of *Staphylococcus* decreased (mean relative abundance in RHM vs. PHM, 27.7% vs. 18.4%)*,* while that of *Streptococcus* and *Pseudomonas* increased (15.8% vs. 24.2% and 7.8% vs. 13.1%, respectively).

## 4. Discussion

In this pilot observational study, we showed that the HM microbiota differs between PTMs and HM donors and provided some insight into the impact of pasteurization on its composition and diversity. In general, our data on the HM microbiota are in line with the available literature. All the most abundant genera we found in the whole cohort (e.g., *Staphylococcus*, *Streptococcus*, and *Acinetobacter*) are in fact already known to be part of the milk core microbiota [31,32]. In particular, the *Staphylococcus* dominance in PT-MOM has already been observed by our research group in a cohort of moderately preterm infants [9], as well as in the other few studies on preterm milk, which report high proportions especially in the early stages of lactation [33,34,35]. It should be remembered that the origin of the HM microbiota is still debated. To date, the microbes present in HM are thought to originate from two different routes, which are probably not mutually exclusive, namely from skin contamination and retrograde flow (salivary backwash) during breastfeeding or, alternatively, through a more speculative gut–mammary route [9,36]. However, most of this knowledge has been derived from mothers of term infants, which may not necessarily be transferable to PTMs [33]. An additional source of bacteria that is certainly relevant for PTMs and their infants is the surrounding environment, including breast pumps and contamination related to milk handling [37]. In the frame of preterm infants’ management, it should be acknowledged that mothers of smaller preterm infants usually do not directly breastfeed but pump their milk that is given to infants via bottle or nasogastric tube, while HM donors usually breastfeed their infants directly; this difference is very likely to impact the HM microbiota features. Since *Staphylococcus* is commonly found in the hospital environment and on the skin of hospitalized preterm infants [38], it is possible that its increased abundance in PT-MOM as reported in this study and others [9,34,35] reflects hospital exposure and direct skin-to-skin contact between the mother or health care professionals and the infant during hospitalization. It should also be noted that a less diverse and *Staphylococcus*-dominated HM community type was more frequently retrieved in the first month after delivery in PTMs who were not fed directly at the breast [9]. Furthermore, PT-MOM was depleted in *Streptococcus*, a typical oral microbe in children [39,40], which was previously found enriched in the term HM [39] as well as being distinctive of a HM community type exclusively associated with samples taken after the infant’s latching to the mother’s breast [9]. It is therefore tempting to speculate that the combination of hospital exposure and limited direct breastfeeding plays a pivotal role in shaping the PT-MOM microbial profiles.

Finally, PT-MOM showed less diversity and smaller proportions of *Pseudomonas* compared with DHM. Regarding *Pseudomonas*, our result is consistent with studies showing a higher relative abundance of *Pseudomonadaceae* in the HM of mothers of term infants who reported using a breast pump compared with mothers who did not [32]. On the other hand, our finding on alpha diversity is in contrast with previous reports, showing similar values between DHM and PT-MOM samples [34,41]. It must be said that the lactation stage has also been shown to have an impact on the HM microbiota [42]. Regarding mother–term infant dyads, several factors including mode of breastfeeding, lactation stage, and maternal body mass index, have been associated with the overall composition of the milk microbiota, although the cumulative association of all the factors assessed in a multivariate analysis explained approximately 25% of the total variation in the milk microbiota composition [32]. On the other hand, only a few studies have assessed the preterm milk microbiota over time using high-throughput sequencing, and based on their results, PTMs showed distinct microbial communities in breast milk that changed across lactation [9,33]. Such temporal changes were often highly individualized, possibly reflecting the intrinsic factors (i.e., transitioning from milk expression and tube/bottle feeding to direct breastfeeding) and peculiar environmental factors that PTMs are exposed to.

With regard to pasteurization, evidence has been accumulating over the last years on the effects of HoP on milk nutrients and bioactive components, but only limited data on the HM microbiota have been reported [20,34,41]. Since it is an effective means of removing any potentially pathogenic bacteria, HoP is thought to most likely kill much of the HM microbiota. However, even if not viable, the bacteria supplied with PHM may still prime the infant immune system and contribute to the establishment of a symbiotic gut microbiota [43,44]. According to our data, HoP affects the HM microbiota structure, with a trend towards greater biodiversity and some compositional differences after pasteurization. In particular, *Streptococcus* and *Pseudomonas* tended to increase while *Staphylococcus* tended to decrease. These data are in agreement with a recent cross-sectional observational study on 42 HM samples from Mexican donors, which found that *Staphylococcus* and *Pseudomonas* were among the major taxa of RHM and PHM, respectively [20]. Furthermore, after heat treatment, the authors observed an increase in bacterial diversity and a greater proportion of some thermoduric bacteria. It should be noted that these differences overlap with what was observed in the comparison between PT-MOM (unpasteurized) and DHM (pasteurized), which confirms the impact of pasteurization/environmental exposure in shaping the HM microbiota. However, it should be acknowledged that the possible non-uniformity of pasteurization practices across experimental settings and differences in heating temperature and pasteurization duration may have an influence on the HM microbiota and its possible paraprobiotic effect.

Some limitations of our study need to be acknowledged. The detection of significant differences between PT-MOM and DHM and mostly between RHM and PHM was limited by the small sample size, and the large inter-individual variation in bacterial composition did not allow us to draw firm conclusions. Furthermore, extensive details on preterm infants’ feeding practices other than direct breastfeeding potentially influencing preterm milk microbiota, such as the use of fresh vs. frozen expressed breast milk given by tube feeding or bottle, the addition of HM fortifiers, variable amount of PT-MOM and DHM feeding, were not available. Additionally, no culture-based characterization of microbiota was performed, affecting our ability to assess the possible viability of HM microorganisms after the pasteurization process.

## 5. Conclusions

HM has proven to be the best feeding option for infants as it relates to better development outcomes both in the short and in the long terms. Its beneficial effects are provided by multiple elements and bioactive compounds, including microorganisms that are attributed a crucial role in the correct priming of immune function. Despite the intrinsic limitations, as discussed above, this pilot study added another piece of knowledge on the HM microbial ecosystem, showing that the DHM microbiota differs from the PT-MOM microbiota, possibly due to inherent differences between HMB donors and PTMs, and that HoP per se impacts the HM microbiota. Given the high importance of exclusive feeding with HM for vulnerable preterm infants, knowledge about the HM microbiota needs to be acquired, in particular by investigating the effect of DHM processing, including but not limited to pasteurization, on the (culturable and unculturable) DHM microbiota and exploring how the latter may affect preterm infants’ gut microbiota development and immune outcomes in this fragile population. In particular, in addition to increasing the sample size, future studies should aim at obtaining viable and quantitative microbiota data to understand whether the infant is exposed to microorganisms (which and how many) or mainly to microbial residues. Not least, other milk characteristics should also be investigated, including pH, nutritional value, proteins, microbial metabolites or components other than DNA. Once this knowledge is obtained, strategies to improve DHM quality while guaranteeing microbiological safety, including the personalization of DHM with PT-MOM inoculum or supplementation with probiotic bacteria isolated from HM or postbiotics may be integrated into clinical practice.

## Figures and Tables

**Table 1:** The clinical and demographic characteristics of mothers who delivered prematurely and mothers recruited as HM donors.

Variable	PTMs (*n* = 7)	HMB Donors (*n* = 14)	*p*-Value
Maternal age, median (IQR), years	32 (28–34)	34 (30.7–36.2)	0.29
Primiparity, *n* (%)	2 (28.6)	2 (14.3)	0.57
Caesarean section, *n* (%)	6 (85.7)	4 (28.5)	0.02
Gestation length, median (IQR), weeks	31.6 (27.9–32.3)	40 (39.4–40.4)	<0.0001

HMB, human milk bank; IQR, interquartile range; PTMs, preterm-delivering mothers.

## Data Availability

The clinical data presented in this study are available on reasonable request from the corresponding author. The microbiota sequencing data are available at NCBI SRA under the BioProject ID PRJNA849176.
